# *Mysidella hoshinoi*, a new species from Izu-Oshima Island, Japan (Crustacea, Mysidae, Mysidellinae)

**DOI:** 10.3897/zookeys.620.9924

**Published:** 2016-09-29

**Authors:** Michitaka Shimomura

**Affiliations:** 1 Kitakyushu Museum of Natural History and Human History, Kitakyushu 805-0071, Kitakyushu, Japan

**Keywords:** Izu-Oshima Island, Mysidae, Mysidella, Sagami Sea

## Abstract

A new mysid, *Mysidella hoshinoi* **sp. n.** is described from Izu-Oshima Island, Sagami Sea, central Japan. This species differs from its congeners in having a posterodorsal finger-like papilla on the eyestalk, five peculiar spines terminating in plumed seta on outer margin of carpopropodus of endopod of first thoracopod, and uropodal endopod bearing 27 spines on inner margin.

## Introduction

*Mysidella* G. O. Sars, 1872, is the only genus of the subfamily Mysidellinae Czerniavsky, 1882 and includes 16 species (WoRMS 2016), ranging in depth from 3 m to 738 m worldwide (Murano 2002). Among these, four species have so far been reported from Japan:

*Mysidella nana* Murano, 1970 at 18–80 m, Oomura Bay, Tateyama Bay, and Suruga Bay (Murano 1970a, 1970b, 2002),

*Mysidella orientalis* Murano, 2002 at 347–369 m, eastern East China Sea (Murano 2002),

*Mysidella tanakai* Ii, 1964 at 220–660 m, Suruga Bay, Tateyama Bay and Sagami Bays (Ii 1964; Murano 1970b, 2002), and

*Mysidella truncata* Murano, 2002 at 138–141 m, Amami-Oshima Island (Murano 2002).

Our recent investigations yielded an undescribed species *Mysidella* from a marine benthic habitat of Izu-Oshima, Sagami Sea. Based on this material, a new species *Mysidella hoshinoi* sp. n. is described, and an updated identification key is provided to the known species of *Mysidella*.

## Material and methods

Mysids were collected with sealable plastic bags (20 cm × 20 cm) by scooping seawater on a sea anemone beloninging to the family Haloclavidae by a local SCUBA diver. All specimens obtained were fixed and preserved in 80% ethanol. Each individual was dissected and prepared for observation by a light microscope (Nikon E600). The total length of individuals was measured from the end of the rostrum to the end of the telson excluding spines.

The terminology follows Murano (2002). The type specimens are deposited in the Kitakyushu Museum of Natural History and Human History, Japan (KMNH).

## Systematics

### Mysidella

Taxon classificationAnimaliaMysidaMysidae

G. O. Sars, 1872

Mysidella G. O. Sars, 1872: 266; G. O. Sars 1879: 84–86; Zimmer 1909: 169; Illig 1930: 600; Banner 1948: 108–109; Tattersall and Tattersall 1951: 427; Ii 1964: 574; Kathman et al. 1986: 191; Fenton 1990: 437; Murano 2002: 66.

#### Type species.

*Mysidella typica* G. O. Sars, 1879 (by original designation and monotypy).

### Mysidella hoshinoi sp. n.

Taxon classificationAnimaliaMysidaMysidae

http://zoobank.org/F7FEE4EB-48E9-4E4A-8AAA-6329D6197FFE

Figs 2 , 3 , 4 , 5

#### Material examined.

Holotype. Adult ♂ (4.0 mm) (KMNH IvR 500893), 34°47'N, 139°24'E, Akino-hama, Izu-Oshima Island, Sagami Sea, Japan, 23 August 2014, 35 m.

Paratypes. Adult ♀ (4.0 mm) (KMNH IvR 500894), immature ♀ (3.0 mm) (KMNH IvR 500895), immature ♂ (3.1 mm) (KMNH IvR500896), data same as holotype; adult ♀ (3.4 mm) (KMNH IvR 500897), immature ♀ (3.0 mm) (KMNH IvR 500898), immature ♂ (2.7 mm) (KMNH IvR 500899), 34°47'N, 139°24'E,Akino-hama, Izu-Oshima Island, Tokyo, Japan, 16 August 2014, 35 m.

#### Diagnosis.

Eyestalk with posterodorsal finger-like papilla; carpopropodus of endopod of first thoracopod with five peculiar spines terminating in plumed seta on outer margin; terminal claw of carpopropodus of endopod of first thoracopod with one short seta and suture distinct; uropodal endopod with 27 spines on inner margin.

#### Description of the holotype.

*Carapace* (Fig. 2A): anterior margin produced into short rounded rostral plate and covering basal part of eyestalks; anterolateral corner produced; posterior margin emarginated, leaving last thoracic somite exposed. Eye (Fig. 2A, C) developed; cornea well-pigmented, globular, wider than eyestalk, occupying nearly half of eye; eyestalk with posterodorsal finger-like papilla.

**Figure 1.**
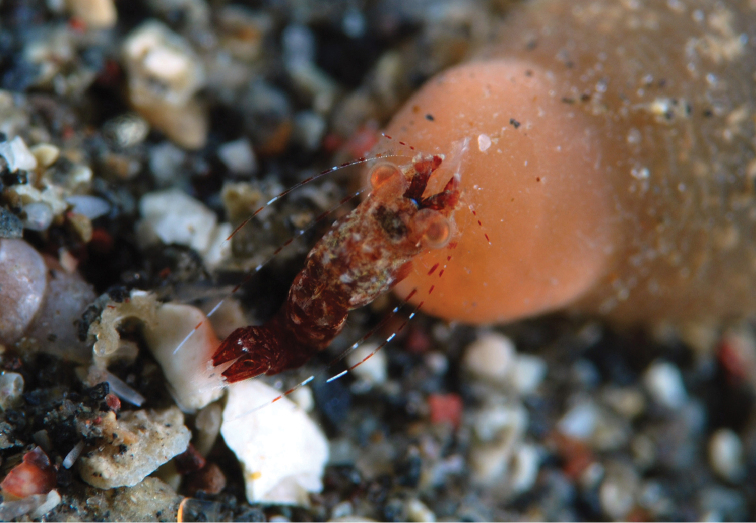
*Mysidella hoshinoi* sp. n., sex unknown, on a tentacle of a sea anemone (family Haloclavidae), Akino-hama, Izu-Oshima Island, Sagami Sea, Japan, 25 March 2016, 35 m depth, habitus *in situ*, photographed by O. Hoshino.

**Figure 2.**
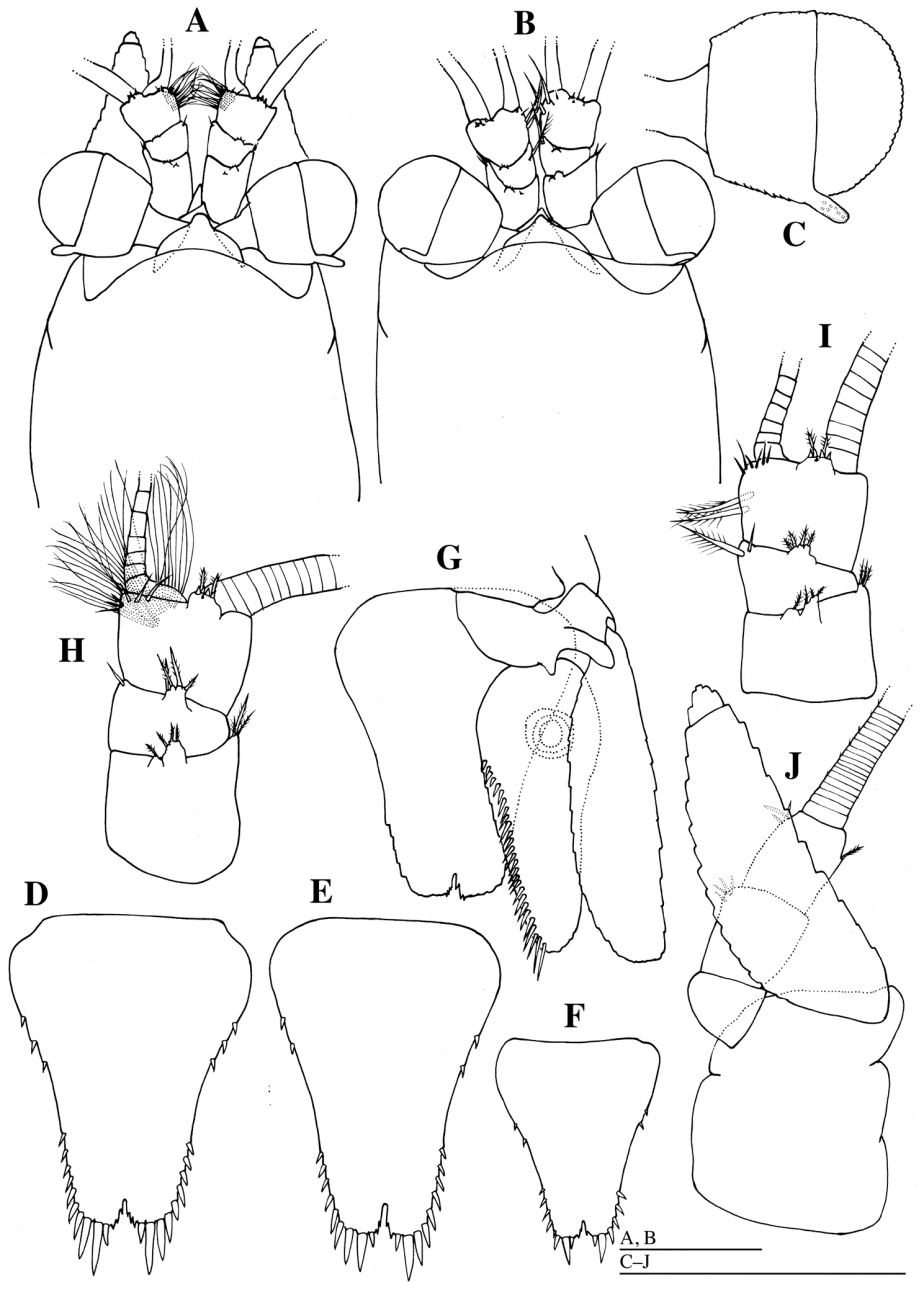
*Mysidella hoshinoi* sp. n., **A, C, D, G, H, J** holotype male **B, E, I** paratype female (KMNH IvR 500894) **F** paratype female (KMNH IvR 500895): **A, B** anterior part of head, dorsal **C** right eye, dorsal **D–F** telson, dorsal **G** telson and left uropod, ventral **H, I** basal part of right antennula, dorsal **J** basal part of right antenna, dorsal. Scale bars: 500 µm.

*Antennula* (Fig. 2A, H): first segment of antennular peduncle longest, 1.3 times as long as third article, with anterolateral corner produced laterally and tipped with three plumose setae, and with two short projections anterodorsally bearing some plumose setae apically; second article shortest, with short projection anterodorsally bearing four plumose setae apically and one simple seta distomedially; third article slightly wider than long, small appendix masculina on ventral side, with short projection anterodorsally bearing some short stout setae and two plumose setae apically, and with six simple setae distomedially.

*Antenna* (Fig. 2A, J): antennal scale setose all round, extending beyond distal margin of antennular peduncle for 0.3 of its length, 3.2 times as long as width, distal suture distinct; outer margin slightly concave; inner margin convex. Antenna peduncle 3-articlulate: first segment shortest; second and third segments subequal in length.

**Figure 3.**
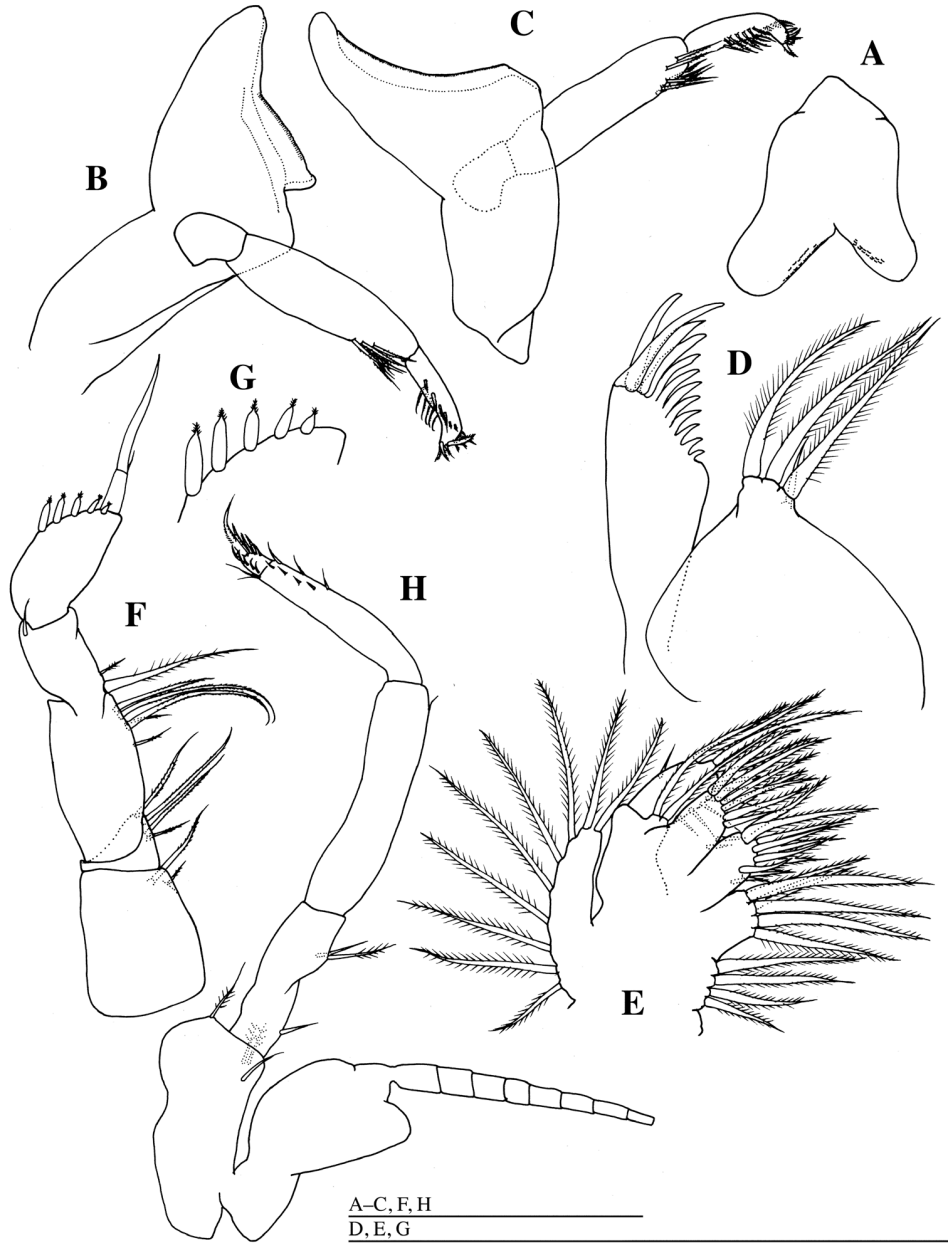
*Mysidella hoshinoi* sp. n., holotype male: **A** labrum ventral **B** left mandible, dorsal **C** right mandible, ventral **D** left maxillula, dorsal **E** left maxilla, dorsal **F** right first thoracopod, dorsal **G** peculiar spines on outer margin of carpopropodus of endopod of first thoracopod **H** right second thoracopod, lateral. Scale bars: 500 µm.

*Labrum* (Fig. 3A) rounded apically, produced posteriorly into two unequal lobes; right lobe broadly rounded posteriorly, with fine teeth on margin; left lobe smaller; both lobes with fine teeth on posterior margin.

*Left mandible* (Fig. 3B) without teeth; molar portion trapezoidal; first article of mandibular palp shortest; second article longest, with seven setulate setae distally; third article slightly curved, with several setae. Right mandible (Fig. 3C) without teeth and molar portion, slightly curved medially, mandibular palp similar in shape than the left one.

*Maxillula* (Fig. 3D): inner lobe broad, 2.6 times as wide as outer lobe, with three plumose and one simple setae; outer lobe with 12 stout setae distally.

*Maxilla* (Fig. 3E): exopod with nine plumose setae on margin; first article of endopod with two plumose setae distally; second article with many plumose and some simple setae on margin; bilobulate basal endites each with ten plumose setae distally; coxal endite with six plumose setae distally and four plumose setae medially.

*Endopod of first thoracopod* (Fig. 3F) robust: basis with two plumose setae; preischium triangular, with four plumose setae distally; ischium 1.1 times as long as basis, with five plumose setae distally; merus 0.6 times as long as ischium, with two plumose setae and one simple seta; carpopropodus 1.4 times as long as merus, twice as long as width, with five peculiar spines (Fig. 3G) terminating in plumed seta on outer margin; terminal claw nearly straight, 1.1 times as long as carpopropodus, with one short setae, suture distinct.

**Figure 4.**
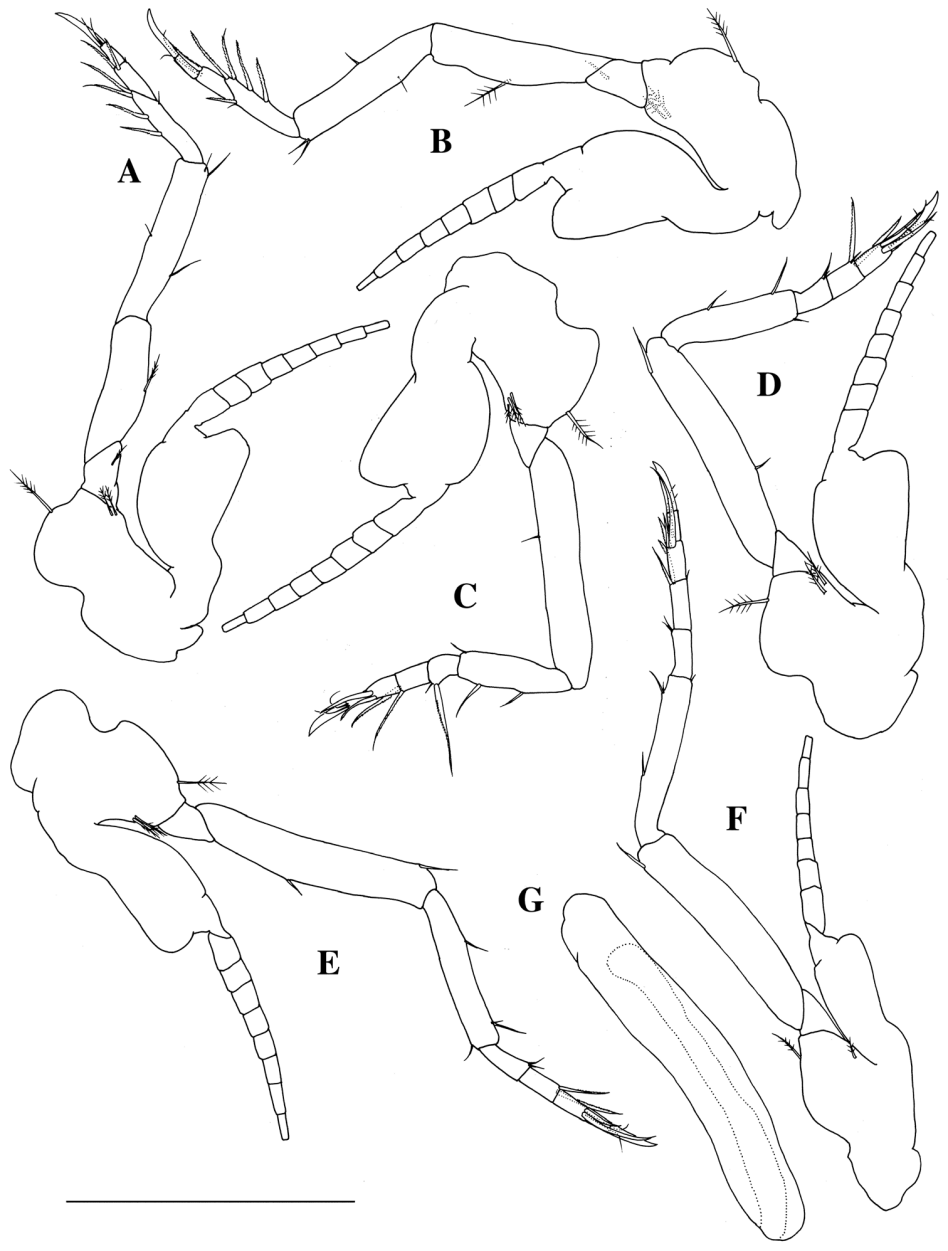
*Mysidella hoshinoi* sp. n., holotype male: **A** left third thoracopod, lateral **B** left fourth thoracopod **C** left fifth thoracopod, lateral **D** left sixth thoracopod, lateral **E** left seventh thoracopod, lateral **F** left eighth thoracopod, lateral **G** left penis, ventral. Scale bar: 500 µm.

*Endopod of second thoracopod* (Fig. 3H): ischium 0.8 times as long as basis; merus longest, 1.6 times as long as ischium; carpopropodus 0.7 times as long as merus, with two rows of setae distally; dactylus small, with one long, setulate seta apically and several short setae. Endopod of third thoracopod (Fig. 4A): preischium trapezoidal; ischium 3.0 times as long as preischium; merus 1.1 times as long as ischium; carpopropodus divided into two subsegments, 0.8 times as long as merus; dactylus small, with strong terminal claw. Endopod of fourth thoracopod (Fig. 4B): preischium trapezoidal; ischium 3.0 times as long as preischium; merus 0.9 times as long as ischium; carpopropodus divided into two subsegments, 0.7 times as long as merus; dactylus small, with strong terminal claw. Endopod of fifth thoracopod (Fig. 4C): preischium triangular; ischium 6.1 times as long as preischium; merus half as long as ischium; carpopropodus divided into three subsegments, 0.8 times as long as merus; dactylus small, with strong terminal claw. Endopod of sixth thoracopod (Fig. 4D): preischium triangular; ischium 5.7 times as long as preischium; merus half as long as ischium; carpopropodus divided into three subsegments, 0.8 times as long as merus; dactylus small, with strong terminal claw. Endopod of seventh thoracopod (Fig. 4E): preischium triangular; ischium 4.2 times as long as preischium; merus 0.7 as long as ischium; carpopropodus divided into three subsegments, 0.8 times as long as merus; dactylus small, with strong terminal claw. Endopod of eighth thoracopod (Fig. 4F): preischium triangular; ischium 3.9 times as long as preischium; merus 0.7 as long as ischium; carpopropodus divided into three subsegments, 0.8 times as long as merus; dactylus small, with strong terminal claw.

*Exopod of first thoracopod with 8-segmented flagellum.* Exopods of second to seventh thoracopods (Figs 3H, 4A–E) similar in shape and size, with 7-segmeted flagellum; basal plate with rounded outer corner. Exopod of eighth thoracopod (Fig. 4F) with 7-segmented flagellum; basal plate narrower than those of anterior six thoracopods.

*Penis* (Fig. 4G) cylindrical, 6.2 times as long as width, without setae.

*Abdomen*: first four somites decreasing in length posteriorly; second and fifth segments subequal in length; sixth somite 1.3 times as long as fifth somite.

**Figure 5.**
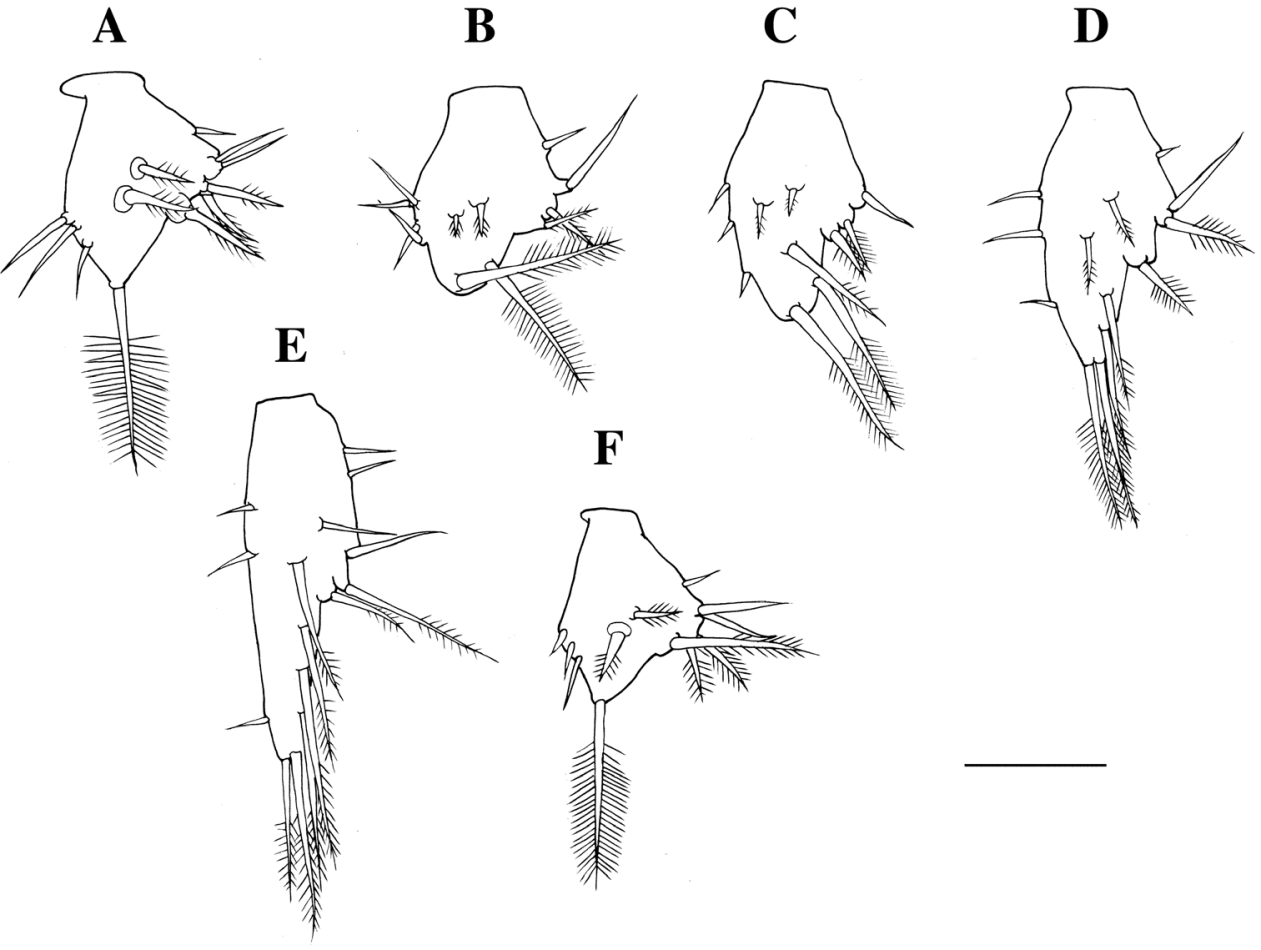
*Mysidella hoshinoi* sp. n., **A–E** holotype male **F** paratype female (KMNH IvR 500894): **A** left first pleopod, ventral **B** left second pleopod, ventral **C** left third pleopod, ventral **D** left fourth pleopod, ventral **E** left fifth pleopod, ventral **F** left first pleopod, ventral. Scale bar 100 µm.

*All pleopods* (Fig. 5A–E) reduced to unsegmented lobe, not modified. First pleopod as long as second pleopod; second pleopod to fifth pleopod increasing in length; fifth pleopod 1.3 times as long as fourth pleopod.

*Uropod* (Fig. 2G): endopod of uropod extending to apex of apical spines of telson, 2.1 times as long as width, with large statolith and 27 spines on inner margin; exopod of uropod 3.9 times as long as width.

*Telson* (Fig. 2D) tapering posteriorly, 1.3 as long as maximum width; lateral margins each with three pairs of anterior spiniform setae, seven posterior spiniform setae on left side and six posterior spiniform setae on right side, and three pairs of terminal spiniform setae; cleft shallow and narrow, 0.08 times as deep as telson length, with six short spines on margin.

#### Description of the paratype female

**(KMNH IvR 500894).** *Antennula* (Fig. 2B, I): first segment of antennular peduncle as long as third article, with anterolateral corner produced laterally and tipped with three plumose setae, and with two short projections anterodorsally bearing some plumose setae apically; second article shortest, with short projection anterodorsally bearing four plumose setae apically and one plumose and one simple setae distomedially; third article slightly wider than long, with short projection anterodorsally bearing some short stout setae and two plumose setae apically, and with two plumose setae medially and six simple setae distomedially.

*All thoracopods and pleopods* (Fig. 5F) similar to holotype male in morphology and chaetotaxy.

*Telson* (Fig. 2E): lateral margins each with two pairs of anterior, six pairs of posterior and three pairs of terminal spiniform setae; cleft with four spines on margin.

*Marsupium* composed of two pairs of developed oostegites on seventh and eighth thoracopods.

#### Variation.

Some variations (N = 7: holotype and 6 paratypes) were recognized in the number of spiniform setae on telson (Fig. 2F). Lateral margins each with two or three pairs of anterior, four to seven pairs of posterior spiniform setae; cleft with one to four spines on margin.

#### Color in life.

Body (Fig. 1) dark to light read, with or without light brownish marbled pattern. Cornea of eye light orange; posterodorsal finger-like papilla on the eyestalk white. Antennular flagella transparent with white and red stripes.

#### Distribution and habitat.

The new species has so far been found only the type locality, 35 m depth, Akino-hama, Izu-Oshima Island, Sagami Sea, central Japan. According to the sampling notes by Mr. O. Hoshino, a number of individuals hovered above and around oral disc and tentacles of Haloclavidae sp. at the bottom. The mysids sometimes perched on the tentacles of the sea anemone. The new species live in ectocommensal association with sea anemones of the family.

#### Remarks.

*Mysidella hoshinoi* sp. n. differs from all the congeners in having a posterodorsal finger-like papilla on the eyestalk.

The arrangement of the spines of the telson links the new species to *Mysidella incisa* Wang, 1998, from the northern area of the South China Sea (Wang, 1998) and the Timor Sea (Murano, 2002). *Mysidella hoshinoi* is distinguished from *Mysidella incisa* by the following characters (those of *Mysidella incisa* in parentheses): cornea occupying nearly half of eye (nearly one third); eyestalk with posterodorsal finger-like papilla (without papilla); uropodal endopod 2.1 times as long as width (2.5–2.7 times as long as width), with 27 spines on inner margin (with 22–24 spines).

#### Etymology.

This species is named after Mr. O. Hoshino, who gave me the present material for taxonomic study. The specific name thus is a noun in the genitive singular.

### Key to the species of *Mysidella*, with the depth ranges and distributions (modified from Brattegard 1973 and Murano 2002)

**Table:** 

1	Eye well developed, with cornea	**2**
–	Eye rudimentary, without cornea. 375 m depth, Norway	***Mysidella typhlops***
2	Posterodorsal finger-like papilla on the eyestalk absent	**3**
–	Posterodorsal finger-like papilla on the eyestalk present. 35 m depth, Izu-Oshima Island, Japan	***Mysidella hoshinoi* sp. n.**
3	Distal cleft / total length in telson less than 5%	**4**
–	Distal cleft / total length in telson more than 5%	**5**
4	Two or three spines on distal cleft of telson. 20–115 m depth, northern South China Sea, Timor Sea	***Mysidella incisa***
–	Six spines on distal cleft of telson. 33–79 m depth, Bass Strait	***Mysidella australiana***
5	Distal cleft / total length in telson less than 10%	**6**
–	Distal cleft / total length in telson more than 10%	**8**
6	Telson 1.3 times as long as width; two to four spines on distal cleft of telson	**7**
–	Telson about twice as long as width; eight spines on distal cleft of telson. 25.5–260 m depth, northern South China Sea	***Mysidella rotundincisa***
7	Three peculiar spines on outer margin of carpopropodus of endopod of first thoracopod; 16 spines on inner margin of uropodal endopod; seven to nine spiniform setae along whole length of lateral margin of telson. 3 m depth, Rottnest Island, West Australia	***Mysidella mukaii***
–	Five peculiar spines on outer margin of carpopropodus of endopod of first thoracopod; 25 spines on inner margin of uropodal endopod; eight spiniform setae on distal half of lateral margin of telson. 138–141 m depth, Amami-Oshima Island, southwestern Japan	***Mysidella truncata***
8	Distal cleft / total length in telson less than 19%	**9**
–	Distal cleft / total length in telson more than 19%	**13**
9	Distal cleft / total length in telson 17%. 500–600 m depth, British Columbia to S. California	***Mysidella americana***
–	Distal cleft / total length in telson less than 15%.	**10**
10	46 spines on inner margin of uropodal endopod	**11**
–	24–32 spines on inner margin of uropodal endopod	**12**
11	Telson 2.4 times as long as width. 300–720 m depth, Bay of Biscay	***Mysidella biscayensis***
–	Telson less than twice as long as width. 415–437 m depth, northern South China Sea	***Mysidella macrophthalma***
12	Six or seven spiniform setae on distal half of lateral margin of telson; 24 or 25 spines on inner margin of uropodal endopod. 40 m depth, Caribbean coast of Colombia	***Mysidella minuta***
–	16–18 spiniform setae on distal half of lateral margin of telson; 30–32 spines on inner margin of uropodal endopod. 90–540 m depth, Norway to Bay of Biscay, Mediterranean	***Mysidella typica***
13	Five peculiar spines on outer margin of carpopropodus of endopod of first thoracopod; 12–20 spines on distal cleft of telson	**14**
–	Three peculiar spines on outer margin of carpopropodus of endopod of first thoracopod; 24–36 spines on distal cleft of telson	**15**
14	20 spiniform setae on distal half of lateral margin of telson; 35 spines on inner margin of uropodal endopod. 80 m depth, Suruga bay, Japan	***Mysidella nana***
–	Eleven or 12 spiniform setae on distal half of lateral margin of telson; 29 spines on inner margin of uropodal endopod. 78 m depth, northern South China Sea	***Mysidella tenuicauda***
15	16 spiniform setae on distal half of lateral margin of telson; 30 spines on inner margin of uropodal endopod. 347–369 m depth, eastern East China Sea	***Mysidella orientalis***
–	25–27 spiniform setae on lateral margin of telson; 47 or 48 spines on inner margin of uropodal endopod	**16**
16	Deep transverse groove on rostrum present; telson 2.6 times as long as width. 535–738 m depth, Timor Sea, Sulu Sea	***Mysidella sulcata***
–	Deep transverse groove on rostrum absent; telson 2.3 times as long as width. 220–660 m depth, Suruga Bay, Sagami Bay, Japan	***Mysidella tanakai***

## Supplementary Material

XML Treatment for Mysidella

XML Treatment for Mysidella hoshinoi
